# Rapid statistical discrimination of fluorescence images of T cell receptors on immobilizing surfaces with different coating conditions

**DOI:** 10.1038/s41598-021-94730-3

**Published:** 2021-07-29

**Authors:** Badeia Saed, Rangika Munaweera, Jesse Anderson, William D. O’Neill, Ying S. Hu

**Affiliations:** 1 Department of Chemistry, College of Liberal Arts and Sciences, University of Illinois at Chicago, Chicago, IL 60607 USA; 2 Department of Chemical Engineering, University of Illinois at Chicago, Chicago, IL 60607 USA; 3 Department of Bioengineering, Colleges of Engineering and Medicine, University of Illinois at Chicago, Chicago, IL 60607 USA

**Keywords:** Biomedical engineering, Fluorescence imaging, Imaging the immune system, Statistics

## Abstract

The spatial organization of T cell receptors (TCRs) correlates with membrane-associated signal amplification, dispersion, and regulation during T cell activation. Despite its potential clinical importance, quantitative analysis of the spatial arrangement of TCRs from standard fluorescence images remains difficult. Here, we report Statistical Classification Analyses of Membrane Protein Images or SCAMPI as a technique capable of analyzing the spatial arrangement of TCRs on the plasma membrane of T cells. We leveraged medical image analysis techniques that utilize pixel-based values. We transformed grayscale pixel values from fluorescence images of TCRs into estimated model parameters of partial differential equations. The estimated model parameters enabled an accurate classification using linear discrimination techniques, including Fisher Linear Discriminant (FLD) and Logistic Regression (LR). In a proof-of-principle study, we modeled and discriminated images of fluorescently tagged TCRs from Jurkat T cells on uncoated cover glass surfaces (Null) or coated cover glass surfaces with either positively charged poly-L-lysine (PLL) or TCR cross-linking anti-CD3 antibodies (OKT3). Using 80 training images and 20 test images per class, our statistical technique achieved 85% discrimination accuracy for both OKT3 versus PLL and OKT3 versus Null conditions. The run time of image data download, model construction, and image discrimination was 21.89 s on a laptop computer, comprised of 20.43 s for image data download, 1.30 s on the FLD-SCAMPI analysis, and 0.16 s on the LR-SCAMPI analysis. SCAMPI represents an alternative approach to morphology-based qualifications for discriminating complex patterns of membrane proteins conditioned on a small sample size and fast runtime. The technique paves pathways to characterize various physiological and pathological conditions using the spatial organization of TCRs from patient T cells.

## Introduction

The advent of single-molecule and superresolution microscopy has enabled the investigation of nanoscale and microscale spatial organization and rearrangement of T cell receptors (TCRs) on the plasma membrane of T cells during T cell activation^1–6^. New mechanistic insights have revealed the distinct roles of TCRs in signal amplification and dispersion^7,8^, distinction between foreign- and self-peptides^2,9,10^, and sensing of mechanical forces^11,12^, among others. It is now known that the clustering of TCRs correlates with some of the functions mentioned above. The spatial redistribution of TCRs and the formation of TCR clusters on the plasma membrane begs the question whether the organization of TCRs, *e.g.*, obtained through standard fluorescence imaging, contain diagnostic or prognostic values. Such information could potentially augment the overall expression level registered by flow cytometry to improve the segmentation and diagnostic accuracy. However, state-of-the-art single-molecule and superresolution microscopy techniques are not yet clinically ready. To fully harness the spatial information of TCR in a statistically significant manner, the imaging modality needs to be relatively high-throughput with reproducible results, both of which currently require substantial development in the single-molecule and superresolution community. Various attempts have been made to analyze TCR distribution^13^ and dynamics^14^ using statistical techniques, yet the lack of rapid TCR cluster analysis techniques using conventional fluorescence images sets the second hurdle. Specialized cluster analysis techniques, such as those developed for single-molecule localization microscopy (SMLM)^15–17^, cannot be readily applied to standard fluorescence images.

Current standard techniques depend on the ability to quantify the size, shape, intensity, and presence of specific phenotypes. Additional biological information contained beyond these standardized characterizations remains difficult to extract without introducing certain bias. For instance, dimensional reduction by principal component analysis^18^ and related techniques^19–21^ requires a priori knowledge of which measured feature is most important in addressing the specific biological question. While data-driven, state-of-the-art artificial intelligence (AI) mitigates such bias, these techniques usually rely on relatively large representative data sets and require dedicated computing power. To this end, a computationally efficient approach is desirable for pilot studies, which can then be optimized and guide the experimental design of “big data” studies.

We set out to develop a rapid, quantitative technique for analyzing standard fluorescence images of TCRs. Our premise is that pixel-based image information contains statistical information useful for discrimination analysis of TCRs from T cell images on different surface conditions. Such strategy has been employed in numerous diagnostic medical image analyses^22–26^. Here, we developed an analytical image analysis technique based on partial differential equation (PDE) image models followed by linear class discrimination of the estimated model parameters. We termed the technique: Statistical Classification Analyses of Membrane Protein Images or SCAMPI. We developed SCAMPI to discriminate Jurkat T cells immobilized on different surface conditions using fluorescence images of TCRs. SCAMPI’s TCR discrimination ability is achieved by employing two discrimination techniques: Fisher Linear Discriminant (FLD) and Logistic regression (LR). The enhanced FLD has been applied in many classification applications, including face recognition^27–29^, speech classification^30^, and hand motion classification^31^, among others. Similarly, LR has been applied in fluorescence image classification^32^, hyperspectral image classification^33^ and image segmentation tasks^34^.

While TCR clustering on activated T cells has been extensively characterized using single-molecule and superresolution imaging, that of non-activated T cells remains an active area of research. Early studies utilized electron microscopy^1,35^, photoactivated localization microscopy^1^, and light-sheet direct stochastic optical reconstruction microscopy (*d*STORM)^4^. More recently, the development of thinning out clusters while conserving stoichiometry of labeling (TOCCSL)^36^ and label-density-variation single-molecule localization microscopy (SMLM)^37^ indicates that the interpretation of the TCR organization and distribution can be affected by the labeling and detection artifacts associated with SMLM. To this end, fluorescence TIRF imaging is immune to the clustering artifact of SMLM. SCAMPI developed towards this imaging modality may circumvent the artifacts that arose from previous imaging studies.

In a proof-of-principle study, we investigated the spatial arrangements of TCRs on the plasma membrane of human Jurkat T cells. We utilized a CD3-EGFP Jurkat E6-1 T cell line and performed total-internal-reflection fluorescence (TIRF) imaging of TCRs. An uncoated glass surface (Null) and two types of coated glass surfaces were used: positively charged poly-L-lysine (PLL) or TCR cross-linking, anti-CD3 antibodies (OKT3). As an inert surface condition, we imaged T cells on a cover glass surface without a ligand, this will be referred to as Null class. PLLs induce nonspecific, electrostatic interactions with the cell membrane and facilitate cell immobilization. We employed a widely-used surface coating condition to immobilize T cells for standard fluorescence imaging^1,2,16,38–43^, although PLL is known to induce partial TCR immobilization and may potentially perturb the T-cell resting state^44–46^. Immobilized OKT3 molecules, the other coated surface condition, stimulate T cells and induce the formation of pronounced TCR microclusters^47,48^. These TCR microclusters have been postulated to serve as signaling units for the proper relay of information and have been associated with the formation of an immunological synapse^1,6,49–52^. While immobilized OKT3 molecules do not result in the formation of the immunological synapse, similar TCR clustering has been observed by other microscopy studies^6,52^. Our goal was to investigate whether SCAMPI is capable of discriminating images from these two surface conditions using a limited number of standard fluorescence images.

## Results

### TIRF imaging of T cells on surfaces coated with different ligands

**Figure 1:**
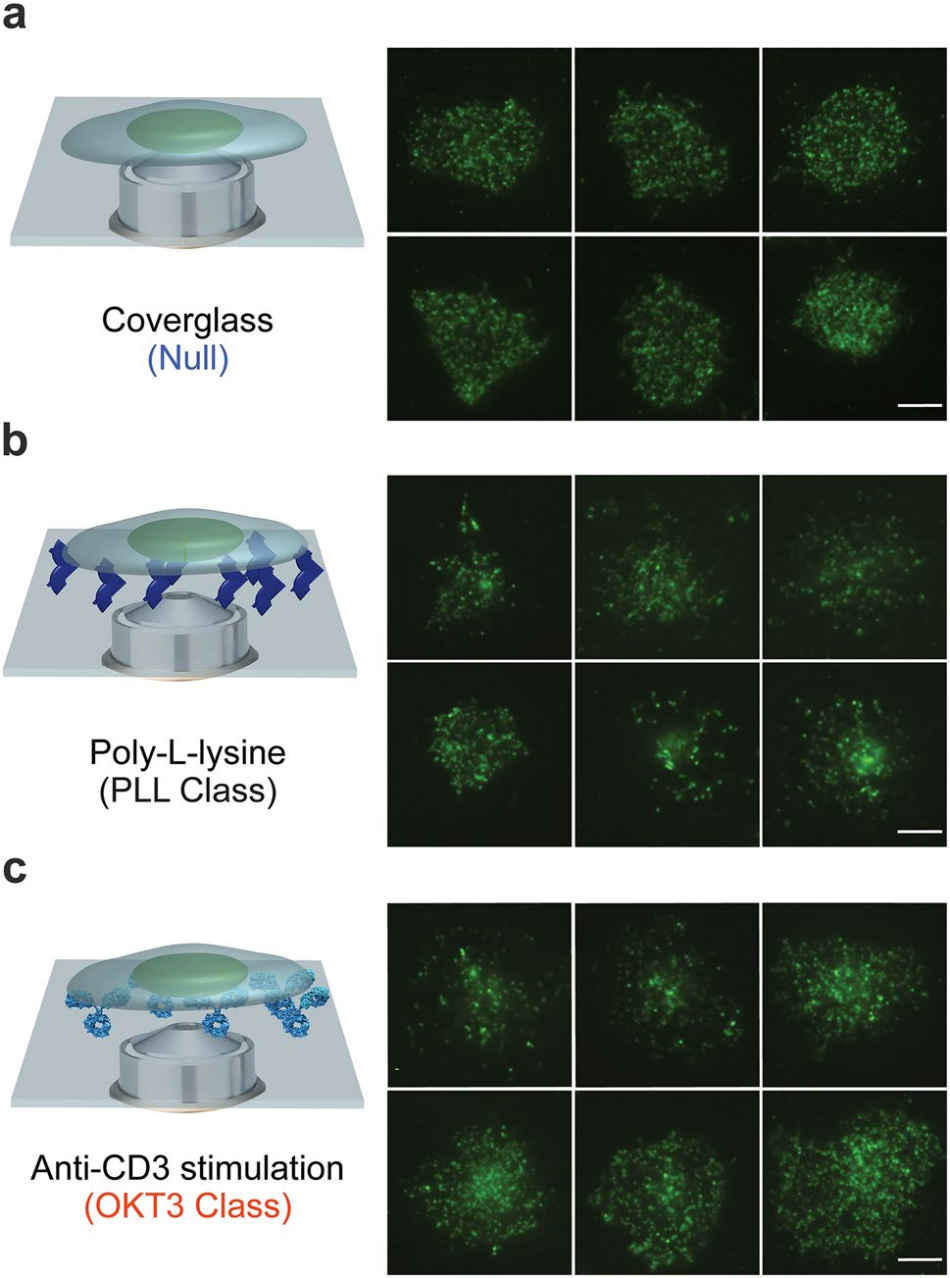
Acquisition of TIRF images of T cell receptors (TCRs) for the development of SCAMPI. (**a**) Null class represents TCR images acquired from an uncoated cover glass. (**b**) PLL class represents TCR images acquired from PLL-coated cover glass. (**c**) OKT3 class represents TCR images acquired from OKT3-coated cover glass. Schematics on the left and representative images on the right. Scale bar: 5 µm.

We obtained high-contrast TIRF images of TCRs from live Jurkat T cells on three types of glass surfaces, which we defined as Null class, PLL class*,* and OKT3 class. Null class depicted TCR images that were acquired from a cover glass surface, without a ligand coating (Fig. 1a, Supplemental Fig. S1). The uncoated cover glass served as an inert surface that would minimally, if at all induce a response in T cells. PLL class represented images acquired from an immobilizing surface coated with PLL (Fig. 1b, Supplemental Fig. S2). The electrostatic interactions between positively charged PLL and negatively charged cell membranes facilitate cell attachment to the glass surface. OKT3 class represented images acquired from the other immobilizing surface, coated with the OKT3 antibody (Fig. 1c, Supplemental Fig. S3). Images were collected by a 100×/1.49 TIRF objective with a 1.5 × external magnification and a Photometric 95B sCMOS camera, resulting in an image pixel size of 73 nm. The 100 images were collected by scanning 2 wells on 4 different days for the PLL surface condition and 5 different days for both OKT3 and Null surface conditions. Calcium imaging data were collected to determine the overall stimulatory effect of the surface condition on the T cells (Supplemental Fig. S4).

### Development of the pixel-based image model for discrimination analysis

**Figure 2:**
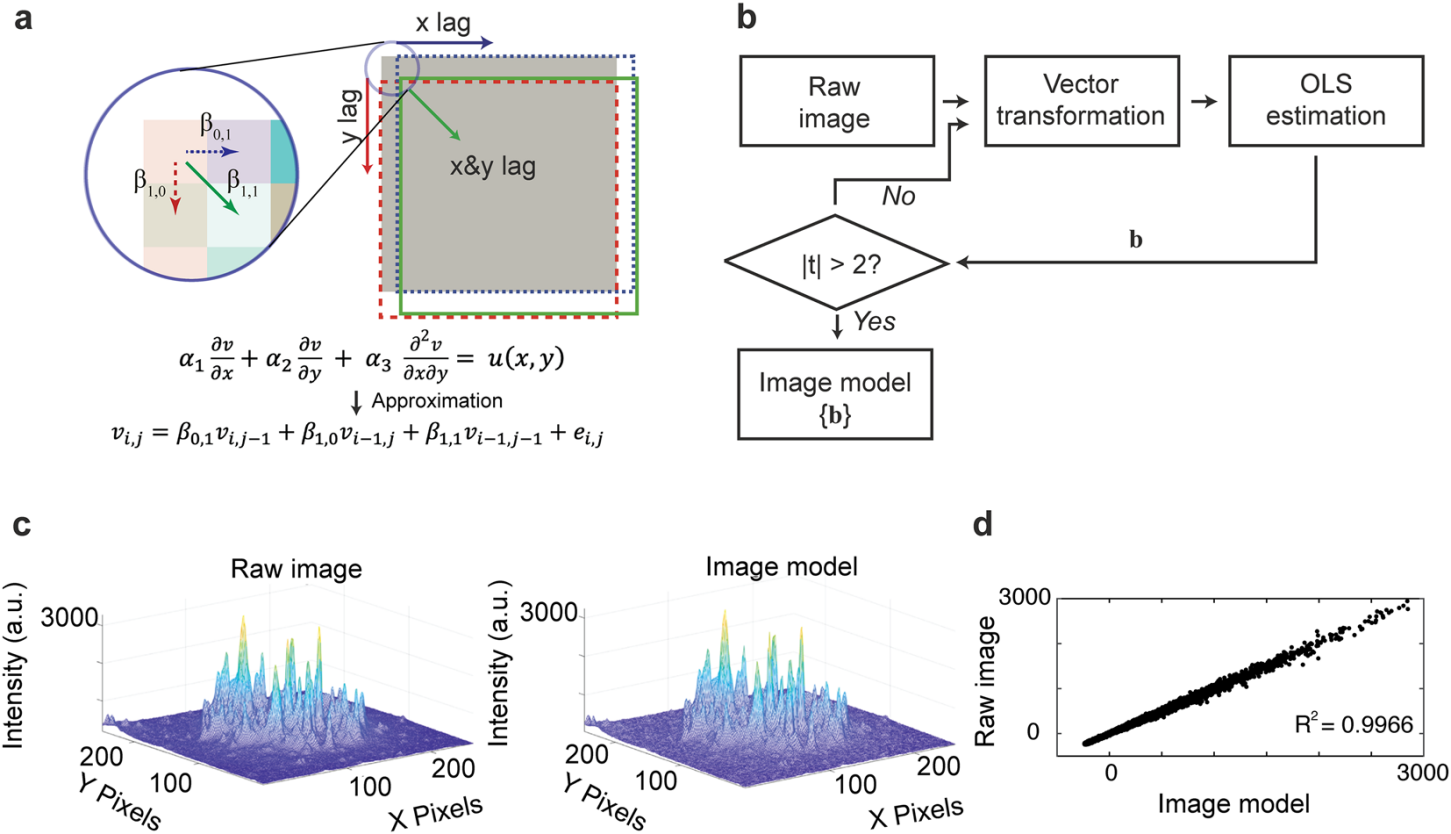
Construction of the fluorescence image model. (**a**) Formulating an image spatial lag structure for the image model and extraction of estimates of representative coefficients (β_1,0_, β_0,1_, β_1,1_) for one spatial lag (**b**) A flowchart outlining the procedures of obtaining image model parameters through ordinary least-square (OLS) estimation. (**c**) A representative intensity profile of a fluorescence image of T cell receptors from a Jurkat T cell on a PLL surface (Raw image) and its OLS image model constructed from a model with 3 parameters (Image model). (**d**) The scatter diagram of the OLS model for a typical TCR image obtained from the PLL surface.

Our image model stems from the observation that the 2-dimensional autocorrelation function of an image is similar to that of a PDE in 2 independent space variables. The original 2D image can be modeled by a sequence of 2D images resulting from pixel shifts, termed spatial lags, in *x* (horizontal shift), *y* (vertical shift)*,* and both *x & y* (horizontal and vertical shifts) simultaneously. Similarly, linear and stationary PDEs contain a linear combination of independent terms described by their defining parameters (Fig. 2a). As such, a linear sequence of images could be modeled as an ordinary least squares regression (OLS) approximation of the PDEs, given that individual images concatenating into a single matrix can be formatted into a linear combination of independent vectors.

To realize this modeling strategy, we first transformed 2D pixel-based fluorescence images into 1D vectors in column-major order: an *m* by *n* pixel image matrix was converted to a *m* × *n*-by-1-pixel vector. A single image regression design matrix is made up of columns, which are spatial lags of the original image. This strategy is a 2D adaptation from time series methods, in which explanatory regression variables represent time lags of the series being estimated. If each image design matrix has *k* columns, then each class has a class parameter matrix consisting of *k* columns and the number of rows equal to the number of images being modeled. We have previously shown this vector transformation preserves the parametric relationship between the PDE model and the image^23^ (Supplemental Note 1).

To identify model parameters in the PDEs, we implemented a general OLS modeling strategy:

$$Image = Image \, Model + Residuals,$$

where the best *Image Model* was obtained by minimizing the variance of the *Residuals*. The design matrix plays a role in the construction of the *Image Model*, and the *Residuals* represents the error in the estimation. We employed Student-*t* tests to evaluate the statistical significance of the model parameters by OLS estimation for each individual image collected. The *t* test used the White asymptotic parameter covariance matrix, as shown in Table S1. In our model construction, “significant” designates a *p* < 0.05 for Student’s- *t* tests and *p* < 0.01 for chi-square tests, unless otherwise noted. If model parameters were found insignificant, spatial lags were increased to reconstruct an alternative image model, until significant model parameters were obtained (Fig. 2b). Model parameters and their Student-*t* statistics were estimated using the detailed method outlined in Supplemental Note 1. For discrimination analyses, training and testing regression models for all images had one spatial lag with 3 significant OLS model parameters (Supplemental Note 1). Figure 2c shows the intensity profile of a typical fluorescence image of the TCRs in a T cell (280-by-280 pixels) and the corresponding image model consisting of a linear combination of three spatial lags (279-by-279 pixels). Figure 2d. shows the fidelity of the image model with an R^2^ = 0.9966. Table 1 shows averaged estimated model parameters of 20 test images from each class. The significant model parameters for each class were found to differ markedly.

**Table 1:** Mean (*n* = 20) model parameters and mean Student-*t* tests of parameters for image models constructed with three parameters (one spatial lag).

	Mean values of OKT3 class cell images			Mean values of PLL class cell images		
	β_0,1_	β_1,0_	β_1,1_	β_0,1_	β_1,0_	β_1,1_
Parameter	0.6443	0.6674	− 0.3438	0.5671	0.5943	− 0.1992
(SD)	(0.0314)	(0.0316)	(0.0541)	(0.0612)	(0.0613)	(0.1043)
[Student-t]	[20.52]	[21.25]	[6.35]	[9.27]	[9.69]	[1.91]

### Fisher linear discriminant of model parameters enables class discrimination

**Figure 3:**
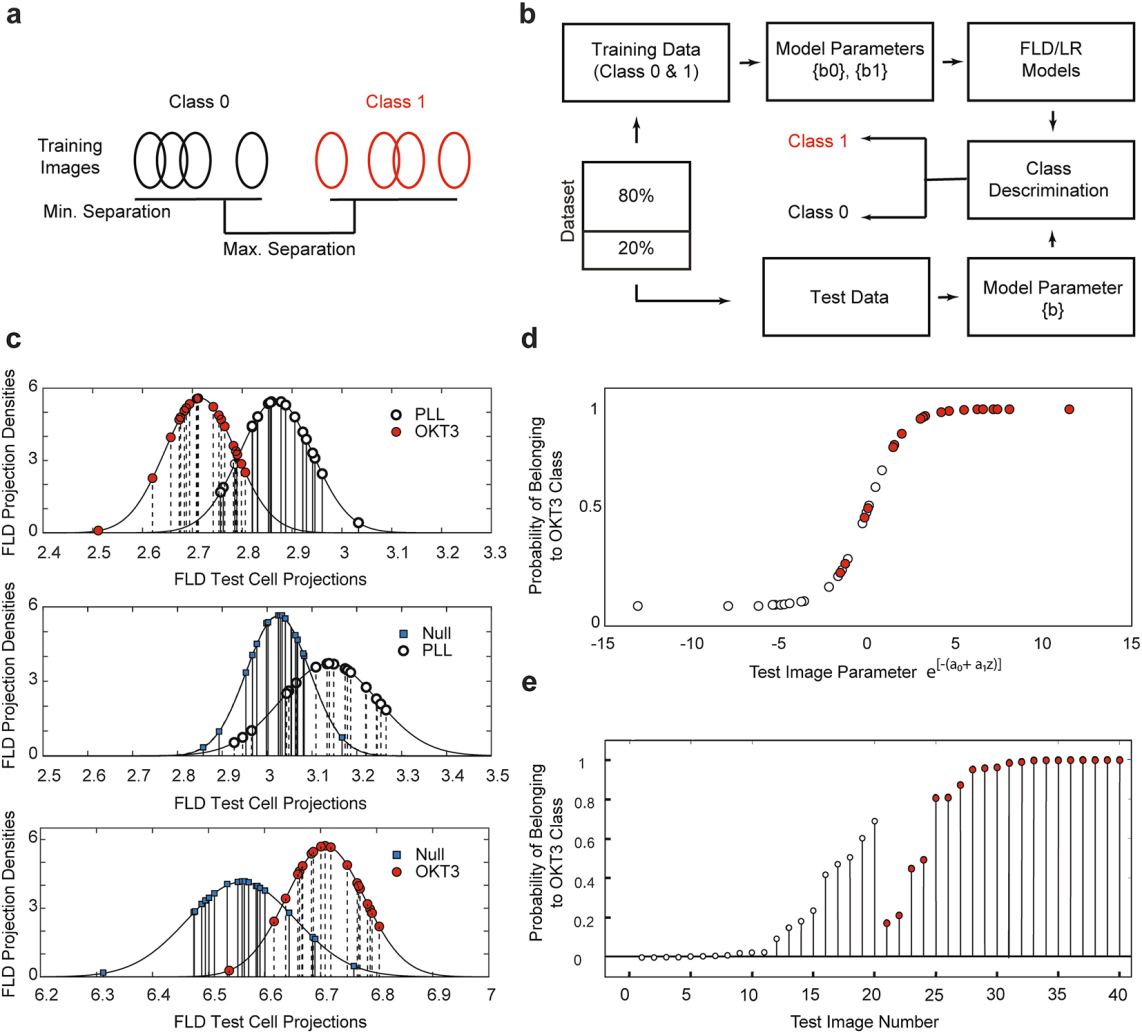
Statistical discrimination of fluorescence images of TCRs using FLD and LR. (**a**) FLD process to maximize the separation between two classes while minimizing intrasample variations. (**b**) The flow chart of SCAMPI development: 80 images were randomly selected as the training dataset and the remaining 20 as the test dataset for each class. (**c**) The 20 OKT3 class (red dots) and 20 PLL class (black dots) average test projections with 85% discrimination; Null class (uncoated cover glass, blue squares) and PLL class (black dots) average test projections with 83% discrimination; Null class (uncoated cover glass, blue squares) and OKT3 class (red dots) average test projections with 85% discrimination. (**d**) The projections from (**c**) are explanatory variables in a logistic regression to estimate the probabilities of class membership in OKT3 class using 20-20 test images from PLL and OKT3 class. (**e**) Probabilities of class membership in OKT3 class of 40 test images in (**d**).

We applied the Fisher Linear Discriminant (FLD) method to achieve class discrimination using model parameters described above. We termed the technique FLD-SCAMPI. Briefly, FLD projects individual parameter vectors as a scalar product. The FLD eigenvalue projection vector allows for maximum separation of the training images from the different surface conditions, for example PLL class and OKT3 class*,* while minimizing the separation within each class (Fig. 3a, Supplemental Note 2). Figure 3b shows the flowchart for FLD-SCAMPI for PLL class and OKT3 class*,* which is similar for discrimination between Null class and PLL class or OKT3 class. We applied FLD to the OLS estimated model parameters from the training dataset containing 80 randomly selected images from these surface conditions*.* The resultant eigenvector for PLL class and OKT3 class was found to be *v*_*c*_ = [1.344 0.797 0.659]^T^; the discrimination of *training* images for this FLD was 76.9%. The cell-to-cell variations and heterogeneities of TCR-specific characteristics among the two classes are accounted for in the discrimination accuracy of these two classes (Supplemental Figs. S2, S3). One hundred OLS image models were estimated for each class. Eighty parameter sets were randomly selected from each image model class as training set data. FLD training set analysis produced the optimal class separating projection vector vc. The dot product of vc and the remaining 20 test image parameter sets were the points on the horizontal axis of Fig. 3c. It can be shown these points are normally distributed as shown on the vertical probability density axis of Fig. 3c.

Figure 3c demonstrates the discrimination of the 20 *test* images not used to construct the FLD eigenvectors. The 3-element projection vector *v*_*c*_ successfully identified 18 out of 20 test images from PLL class and 16 out of 20 test images from OKT3 class. The overall class discrimination of the test images was 85%. A discrimination accuracy of 85% can also be seen between OKT3 class and Null class. Between Null class and PLL class, the discrimination was 83%, where 14 out of 20 test images from PLL class were correctly identified, and 19 out of 20 Null test images were correctly identified by SCAMPI. FLD-SCAMPI is subject to the small sample test of a linear discriminator. We found that smaller sample sizes and overfitting of the training set can lead to decreasing discrimination efficiencies. Additionally, we randomly selected 20 OKT3 images from the first 50 images and 20 from the second 50 images for the FLD-SCAMPI, as a test to determine whether SCAMPI can discriminate between the same class. The resulting FLD showed a classification error of 37.5% (Supplemental Fig. S5). The Kullback minimum discrimination information statistic failed to support at the 5% level the hypothesis that distributions of these two sets of images are different. The result suggests the robustness of our technique, and that FLD-SCAMPI does not discriminate between two non-overlapping subsets we randomly formed within the same class. Notably, the total runtime for the entire analysis, including image data download, model construction, and image discrimination, was collectively 21.89 s on a laptop computer. On average, it took 20.43 s to download the image data, 1.30 s for FLD-SCAMPI, and 0.16 s to run LR-SCAMPI. We attribute the minimal computational footprint to the analytical form of our statistical model and image-derived statistics for the classification.

SCAMPI is sensitive to other changes as well, such as cell spreading. In order to investigate cell spreading on the different surface conditions, we determined the distribution of the surface area of the cells using a MATLAB code that labelled individual pixels in each image of the specific class using a binary system: 0 (below specified threshold) and 1 (above specified threshold). The pixels with intensities greater than the specified threshold were used to determine surface area of cell spreading (Supplemental Fig. S6). While an up-shift was observed from images obtained on the OKT3 surface (Supplemental Fig. S6), differentiating an individual image whose intensities in the overlapping region (< 2 × 10^4^, a.u.) remains challenging using traditional approaches. Additionally, we examined the averaged fluorescence intensity of TCR images. An up-shift of intensity can be observed from the OKT3 distribution, which is in alignment with the fact that OKT3 induced more TCR clustering, and thus higher fluorescent intensity values. Despite this trend in the intensity distribution, only 19% of the OKT3 images displayed intensities brighter than 2.5 × 10^7^ (a.u.), the maximal intensity registered by the PLL images. As such, all 100 PLL images and 81 OKT3 images out of 200 total images cannot be differentiated from each other based on the intensity threshold. In comparison, our SCAMPI achieved substantially better results with 85% classification accuracy (Supplemental Fig. S7). In addition, the more pronounced clustering of TCRs may shift the distribution of intensities registered as pixel values upwards from individual images. Such change may be sufficient to discriminate images from PLL versus OKT3 and NULL versus OKT3. We extracted signals as pixel values greater than 1.1 times the median intensity of the image as background. We employed lognormal parameter estimates to extract the mean (µ) and standard deviation (σ) from the distribution parameters of signals from each image. Supplemental Fig. S8 revealed a significant overlap between PLL versus OKT3 and NULL versus OKT3, demonstrating the better separation achieved by our SCAMPI in comparison.

### Logistic regression estimated class probabilities

The FLD projections from the previous section represent a weighted sum, or scalar product, of the image’s model parameters. It is important to note that the FLD-based SCAMPI (Fig. 3c) does not give the probability that a given image belongs to a particular class. To overcome this limitation, we developed SCAMPI using Logistic Regression (LR), or LR-SCAMPI.

LR-SCAMPI estimates the probability of each test image belonging to the OKT3 class*.* To this end, we employed a logistic function to model the binary dependent model parameters between PLL class and OKT3 class*.* Test images from PLL class are expected to have low probabilities of being classified into OKT3 class (close to 0); whereas test images from OKT3 class are expected to have high probabilities (close to 1). To estimate the cell probabilities, the classified cell projections (the independent axis entries in Fig. 3d) become the independent variable in a logistic regression. The dependent variable estimated is the LR estimation of each cell’s probability of belonging to OKT3 class. LR-SCAMPI outputs a value (y axis in Fig. 3d) as the probability that an image was obtained from the OKT3 surface. A value closer to 1 represents that the image is mostly likely obtained from the OKT3 surface and a value closer to 0 indicates the image is most likely obtained from the PLL surface. This double optimization is possible because SCAMPI is less susceptible to the statistical limitations that affect popular AI techniques. We provide the technical details of the logistic regression in Supplemental Note 3 and associated Supplemental Fig. S9. We show that the SCAMPI optimization is essential for probability estimation, as the independent variables of the LR parameters are not sufficient alone for probability estimation.

In addition to the similar classification results obtained by FLD-SCAMPI, LR-SCAMPI acts as a corroboration of the FLD (Fig. 3c). An examination of Fig. 3d,e indicates 25 out of 40, or 62.5%, of the test images are within 10% of probability 0 or 1.0. The results also confirmed the consistency between the FLD and LR discrimination techniques. The ability to classify between fluorescent TCR images from PLL and OKT3 surfaces suggests the feasibility of statistical quantification of TCR clusters using pixel-based values from fluorescence images.

## Discussion

Our results indicate that pixel-based image information contained clustered features that can be classified into similar groups. These features could be contributed by the clustering state and global distribution of TCRs upon contact with different ligands. In addition, we observed that T cells flattened more on the OKT3 coated surface upon interaction. This larger degree of cell spreading could have also played a role in the differentiation between Null or PLL class and OKT3 class.

The effectiveness of FLD-based SCAMPI may depend on both the spatial distribution and fluorescent intensity of individual TCR clusters. The FLD projection of an individual cell represents an optimal weighted average of that cell’s OLS parameters, also known as FLD eigenvector weights. To this end, our model parameters are sensitive to the image format and quality. Such dependence can be minimized by collecting images under identical experimental conditions. Unique characteristics related to the optical system, sample preparation, and data acquisition have been normalized within these imaging data. Image characteristics, including the point spread function of the optical system, higher-order optical aberrations, sample labeling densities, photophysical properties of different fluorescence labels, pixel size, and quantum efficiency of the detector camera, can play critical roles in the classification accuracy. In addition, the expression level of EGFP-tagged TCRs may also affect the results. In this case, immunofluorescence staining may be used.

We expect that pre-screening of images for quality control, such as for those obtained in clinical settings, could further improve the discrimination accuracy; however, small fluctuations in the data set may be tolerated by SCAMPI. For instance, out of the 100 PLL class images (Supplemental Fig. S2) whose average background intensity ranged from 127 to 180 (a.u.), four images had a high background intensity in the range of 193–294 (a.u.). The fluctuations may be due to user error associated with the TIRF angle, the set exposure, or laser intensity. These four images were included as part of the training data set for constructing the FLD projection. Despite the intensity fluctuations, these four images accounted for only 5% of the training data set. Therefore, their impact on the FLD projection and subsequent discrimination is attenuated. Such characteristic renders SCAMPI immune to small fluctuations.

Using FLD and LR, we demonstrated rapid classification using only 100 images from each class. We attribute the unique capability of SCAMPI to two salient factors. First, the vector transformation of fluorescence images provides the OLS regression analyses with a large degree of freedom (for example, the three-parameter model of Fig. 2c has 25,761 samples per estimated parameter). As such, the subsequent discrimination analyses leverage robust image-derived statistics. Second, the OLS estimation minimized inter-system noise. The PDE image model not only carries information about the number of TCR clusters and characteristics, such as their size and shape but also the detailed spatial structure of the image. Each training image positively enhances the class discrimination model. In our demonstration, as few as 20 test images per class were found to be sufficient in achieving significant class separation and probabilistic corroboration. However, over-fitting may occur and degrade class separation by SCAMPI, resulting in less significant classification of images due to the random characterizations within the data set itself. FLD-SCAMPI is subject to the small sample test of a linear discriminator. We found that smaller sample sizes and overfitting of the training set can lead to decreasing discrimination efficiencies. For example, using FLD projections, the discrimination of a 40–40 test image set of PLL class and OKT3 class, comprised of 20 random training images and the original 20 test images, yielded a moderate discrimination of 72.5% compared to the 85% of the 20–20 test image set of those two classes. This lower discrimination accuracy may be a result of data overfitting, representing a limitation of SCAMPI. Nevertheless, a unique advantage of our technique lies in the rapid discrimination using small datasets. In our study, an OLS model estimate was achieved in 4.4 ms and the entire model construction and image classification took less than 22 s. One potential application of SCAMPI is the rapid screening of a large combination of experimental conditions and data sets. The pilot results can then guide and optimize the experimental design of more data-demanding investigations. In this study, we employed controlled surface conditions to develop and validate the SCAMPI technique. In addition to the surface ligands, SCAMPI can be applied to time point studies, where TCR organizations may display unique temporal dynamics. Applications of SCAMPI to physiological samples require further considerations owing to the complexity and heterogeneity of single cells.

Our 85% discrimination accuracy translates to the correct identification of 85% of all images obtained from PLL *vs* OKT3 or OKT3 *vs* Null surfaces. In addition to the discrimination accuracy, the “in-between” data points from LR-SCAMPI (Fig. 3d) may provide additional information. In the clinical context, these data points may indicate the requirement of further evaluation or testing. Such information can potentially mitigate the risk of misdiagnosis, but it is not captured in binary classification or reflected by the classification accuracy. In the future, such capability can enable the evaluation of the health status of patient T cells before initiating T-cell-based therapies. For drug development, SCAMPI may be effective in observing the efficacy of T cell inhibitor drugs that prevent the formation of TCR clusters^53^.

SCAMPI may accelerate the translation of mechanistic understanding obtained by cutting-edge fluorescence microscopy^4,54–56^ into clinical applications. As we demonstrate in this proof-of-principle study, determining whether a given fluorescence image of TCRs corresponds to OKT3, PLL, or inert surfaces can be difficult due to nuanced differences and heterogeneity displayed by the images (Supplemental Figs. S1, S2, S3). Image analysis techniques used to differentiate clustering states of TCRs has largely remained in the domain of low-throughput single-molecule and superresolution microscopy studies. In parallel, lack of interpretability and prediction-oriented algorithm design presents a fundamental problem for machine learning and deep learning-based discrimination strategies on standard fluorescence images. In contrast, the SCAMPI model development entirely depends on the relationship between the variables and the significance of the relationship. With its fast run time, SCAMPI can drive the development of high‐resolution and high-throughput imaging flow cytometry to improve diagnostic accuracy by incorporating the spatial distribution of membrane markers.

In this report, we demonstrate a computationally efficient statistical technique to discriminate fluorescence images of TCRs from Jurkat T cells on uncoated glass surfaces and coated surfaces with different ligands. SCAMPI is computationally effective using a small sample size. Our proof-of-principle study using TCRs indicates their global distributions, in addition to the clustering state, may contain physiologically relevant information. Such information will complement single-molecule and superresolution studies to reveal the impact from the heterogeneous distribution of TCRs and associated membrane proteins on the regulation of TCR signaling and downstream T cell function. The successful demonstration of SCAMPI suggests that pixel-based image information can be utilized to classify complex organizations of membrane proteins beyond standard quantification techniques for fluorescence images. In the future, SCAMPI can be extended to study cells interacting with agonist versus non-agonist peptide-major-histocompatibility-complexes and other membrane markers^51^, as well as with different imaging modalities^57^, creating inroads for transforming fluorescence image-based discovery into clinical applications.

## Methods

### Cells and reagents

Jurkat E6–1 T cells (ATCC TIB-152) that express CD3ɣ -GFP were cultured in RPMI 1640 Medium (Gibco, USA, CAT#: 11875119) supplemented with 10% Fetal Calf Serum (FCS) (Gibco, USA, CAT#: 14190-149) and 1% Gibco Penicillin Streptomycin (Thermo Fisher Scientific, CAT# 15140-122) in a 5% CO_2_ humidified atmosphere at 37 °C. Cells were incubated on the immobilizing surface in growth medium for 30 min prior to imaging. The growth medium was replaced with pre-warmed Imaging Buffer consisting of HBSS (Life Technologies, USA, CAT#: 14175-095) supplemented with 1% FCS right before imaging. For cells imaged on the cover glass surface, cells were incubated in growth medium without FBS for 30 min at 37 °C and 5% CO_2_, then growth medium was replaced with pre-warmed imaging buffer without 1% FCS. Monoclonal antibody against CD3ε (clone: OKT3, CAT#: BE0001-2-25MG) was purchased from Bio X Cell, USA.

### Surface preparation

Eight-well chamber cover glasses (Borosilicate sterile No 1.5, CAT# 155409, Lab-Tek) were cleaned with absolute ethanol and dH_2_O, then incubated overnight at room temperature. Stimulating surfaces were produced by adding OKT3 antibody (200 μL) at a concentration of 1 μg/ml in PBS (from Gibco, USA) into a well. Poly-L-lysine (PLL) surface were produced by adding PLL (200 μL) at a concentration of 0.01% in H_2_O (P8920 from Sigma-Aldrich, CAS#: 25988-63-0) into another well. Eight-well chamber slides containing OKT3 and PLL were incubated overnight at 4 °C. For the inert surface, a room temperature, sterile chamber slide was used.

### Live imaging of TCR clusters

Supernatants of the wells containing OKT3 and PLL were decanted, and cells (50-100 k) in culture media were added to each well. They were incubated for 30 min at 37 °C and 5% CO_2_. After the incubation, cells were observed under a conventional microscope to confirm whether they were attached to the surface. Supernatants of the wells were decanted, and pre-warmed imaging buffer was added to the wells; cells were imaged live. The 100 images were collected by scanning 2 wells on 4 different days for the PLL surface condition and 5 different days for both OKT3 and Null surface conditions.

### Total internal reflection fluorescence (TIRF) microscopy

TIRF microscopy experiments were performed on a Nikon Eclipse Ti2 inverted microscope equipped with a 100×/1.49 oil-immersion objective and a 1.5 × external magnification. For TIRF imaging, 488 nm laser was used. Emission light was filtered using appropriate filter sets and recorded on a Prime 95B sCMOS camera with a pixel size of 73 nm in the image plane. Images of TCR clusters were acquired with 2.15 mW (20%, 488 nm) laser power at a 200 ms exposure time.

### SCAMPI standard model statistics

The standardization proposed in the following model is based on the diffusive and advective structures currently reported and studied in the cell model literature^58,59,60^. For this purpose, we propose the PDE model in (1), which is a temporal equilibrium form of a nonhomogeneous, hyperbolic PDE (Supplementary Note 1). Its digital, estimable form in (2) illustrates the model dependence on protein advection parameters and diffusion parameters.

1 $$\alpha_{1} \frac{\partial v}{{\partial x}} + \alpha_{2} \frac{\partial v}{{\partial y}} + \alpha_{3} \frac{{\partial^{2} v}}{\partial x\partial y} = u\left( {x,y} \right)$$

2 $$v_{i,j} = \beta_{0,1} v_{i,j - 1} + \beta_{1,0} v_{i - 1,j} + \beta_{1,1} v_{i - 1,j - 1} + e_{i,j}$$

The regression models for both training and testing utilized spatial lags, providing 3 OLS parameters. The spatial lags were in the x, y, and both x and y direction. After 3 parameters (one spatial lag), no training images had additional significant parameter estimates, therefore the test images were projected by 3 by 1 eigenvector. This projection vector was applied to the 40 test images (20 from each class).

To meet the demands placed on (2) as a T cell protein membrane model, the number of images for estimating the parameters is restricted so that parameter significance is maintained to support an accurate discriminator as well as an accurate predictor of individual class member probabilities. Overfitting degrades parameter significance and compromises discrimination, which in turn compromises estimation of individual class member probabilities.

To determine the overall effect, in training and testing classes, that overfitting has on image discrimination, 20 images from PLL class and OKT3 class were selected randomly as a FLD training set. The separation of the 20–20 images resulted in a mean discrimination accuracy greater than 85%, as noted in Table 1; however, when this same procedure was followed with 80–80 images, the mean discrimination accuracy was 76.9%. This decrease in discrimination accuracy as a direct outcome of an increase in training images, coincides with the hypothesis that overfitting will degrade parameter significance and compromise image discrimination. Because the OLS regressions each have a very large degree of freedom, parameter significance is degraded by increased parameter numbers and image sample numbers from over fitting.

An important assumption of our statistical model is that the image parameters are normally distributed. All 20 element b parameter vectors for both classes passed a Kolmogorov–Smirnov test for normality at 0.05 or better (Table 1). Fig. S3 confirms, in the sense of McShane and Gal^61^, the normal distribution of estimated b_k,l_ values.

The Student-*t* statistics in Table 1, as noted, were computed using the White asymptotic parameter covariance matrix.

### Calcium imaging

Fura-2 AM was freshly thawed in cell culture media to a final concentration of 5 µM. T cells were incubated in the Fura-2 AM solution for 50 min in the dark at room temperature in a 1.5 mL microcentrifuge tube. Cells were washed once with HBSS, then incubated for 10 min in HBSS at room temperature in the dark. T cells were then resuspended in their corresponding imaging buffer.

Cells were diluted using respective imaging buffers and added to wells containing their specific surface conditions (PLL, OKT3, Cover glass).

Cells were imaged with a Nikon Eclipse Ti2 inverted microscope with a 40×/1.30 oil-immersion objective and a 1.5 × external magnification. A Retrapad that can trigger between 340 and 380 nm excitation wavelengths was used. 10-min videos were taken using 50 ms exposure time, 1 s time delay, and 2 × 2 binning.

### Determining effect of cell spreading

The distribution of the surface area of the cells was completed using a MATLAB code that labelled individual pixels in each image of the specific class using a binary system: 0 (below specified threshold) and 1 (above specified threshold). The pixels with intensities greater than the specified threshold were used to determine surface area of cell spreading.

### SCAMPI GitHub repository

Our 100 fluorescent images from the three different surface conditions and the MATLAB codes we used have been deposited on GitHub at the following address: https://github.com/jesse-anderson/The-Ying-Hu-Group.

## Supplementary Information

Supplementary Information.

## Data Availability

Image data and MATLAB codes used in this work have been deposited to the following GitHub address: https://github.com/jesse-anderson/The-Ying-Hu-Group.
