# Supplementary material for: Rapid statistical discrimination of fluorescence images of T cell receptors on immobilizing surfaces with different coating conditions
Source: Sci Rep. 2021 Jul 29;11:15488. doi: 10.1038/s41598-021-94730-3 (PMC8322097; doi:10.1038/s41598-021-94730-3)
Supplement: Supplementary file 1 — Supplementary Information. [file 41598_2021_94730_MOESM1_ESM.pdf]

Supplemental Information

Rapid statistical discrimination of fluorescence images of T cell receptors on immobilizing surfaces with different coating conditions

Badeia Saed[1], Rangika Munaweera[1], Jesse Anderson[2], William D. O'Neill[3,*], and Ying S. Hu[1,*]

[1] Department of Chemistry, College of Liberal Arts and Sciences, [2] Department of Chemical Engineering, [3] Department of Bioengineering, Colleges of Engineering and Medicine, University of Illinois at Chicago, Chicago, IL 60607

[*] woneill@uic.edu; yshu@uic.edu

List of content

Supplemental Fig. S1-9
Supplemental Note 1-3

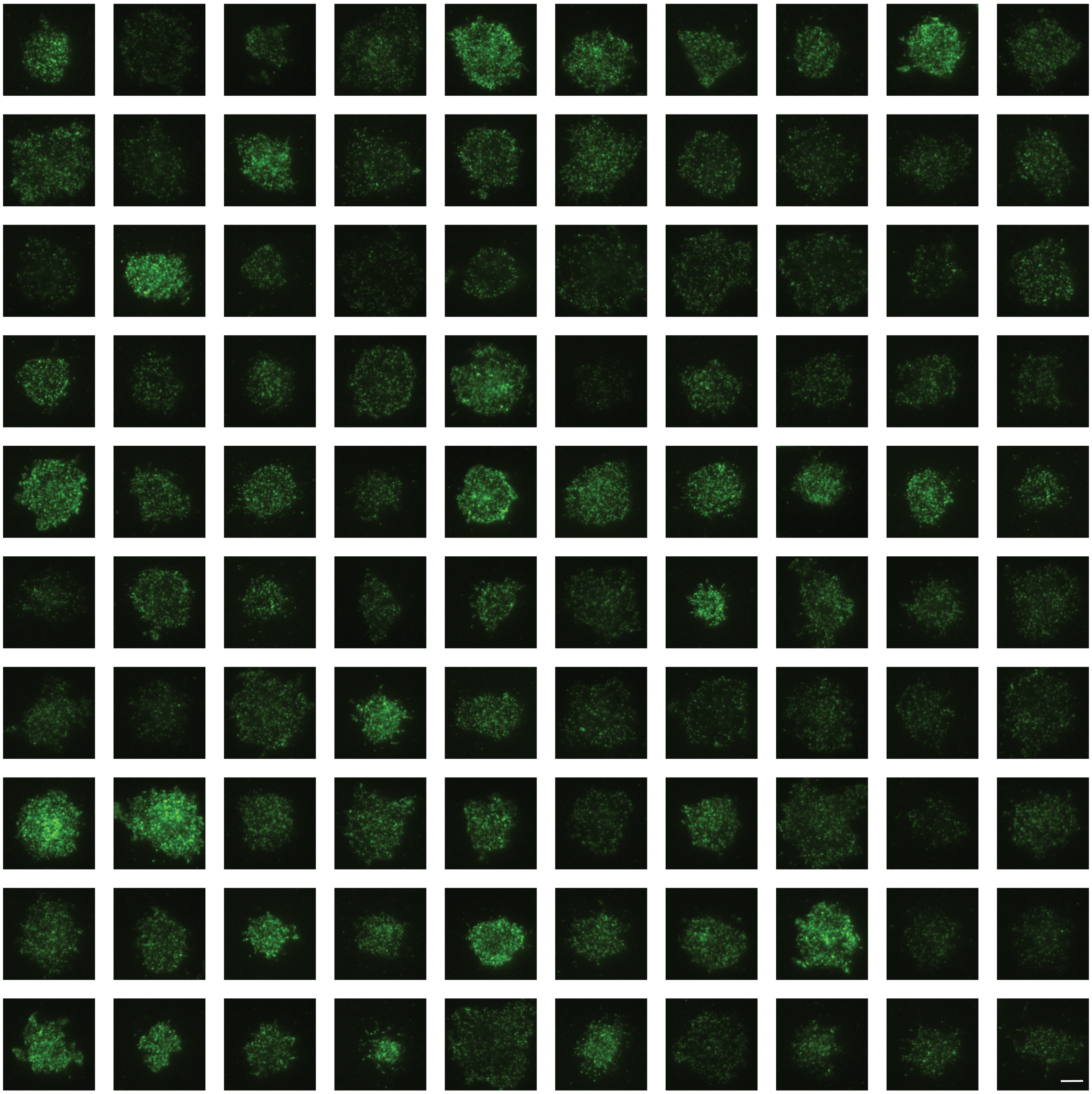

Supplemental Figure S1. All 100 TCR images from the coverglass surface (Null) used to develop SCAMPI. All images were displayed in the same contrast. Scale bar: 5 µm.

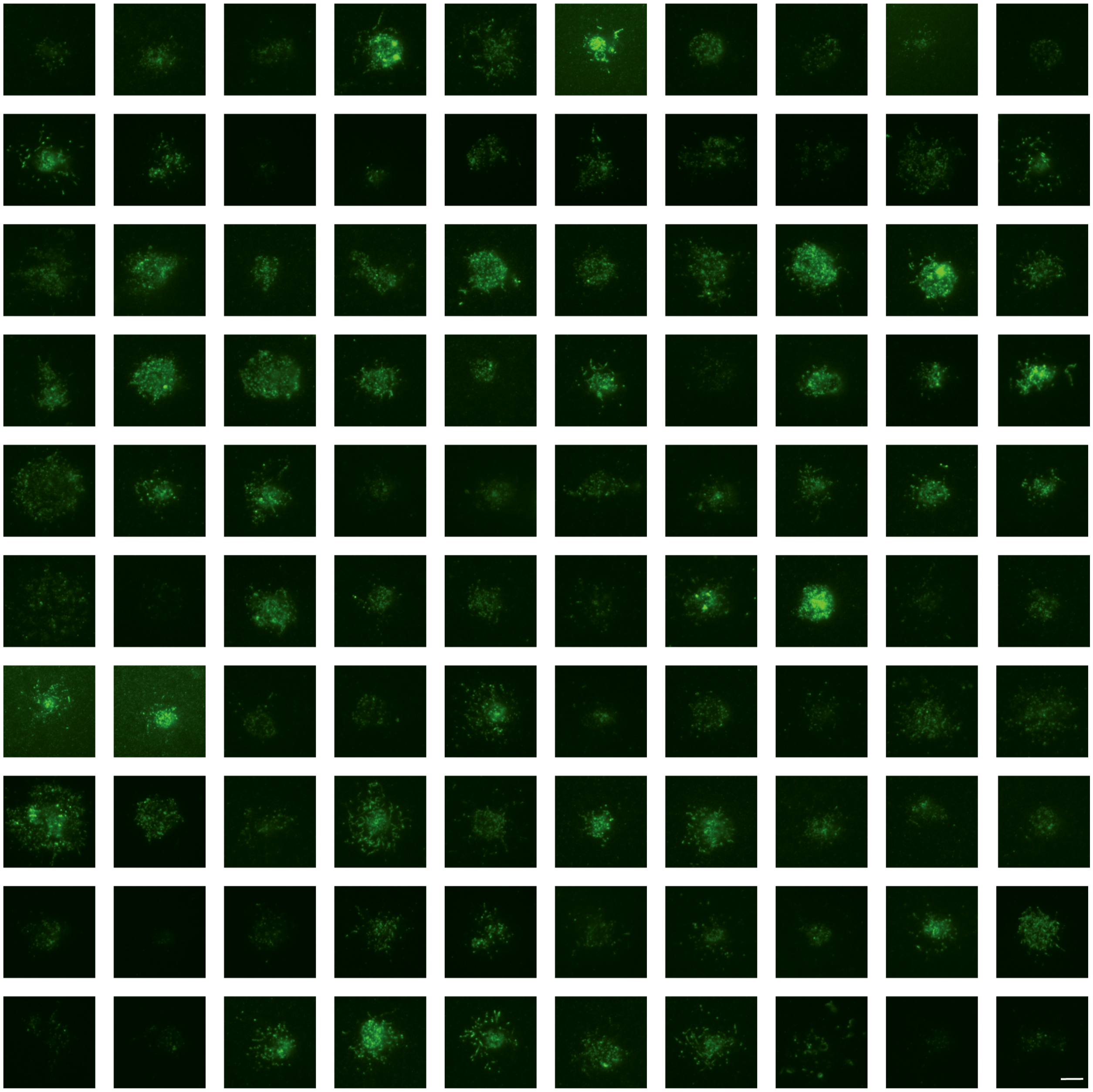

Supplemental Figure S2. All 100 TCR images from the PLL surfaces (*Class 0*) used to develop SCAMPI. All images were displayed in the same contrast. Scale bar: 5 μm.

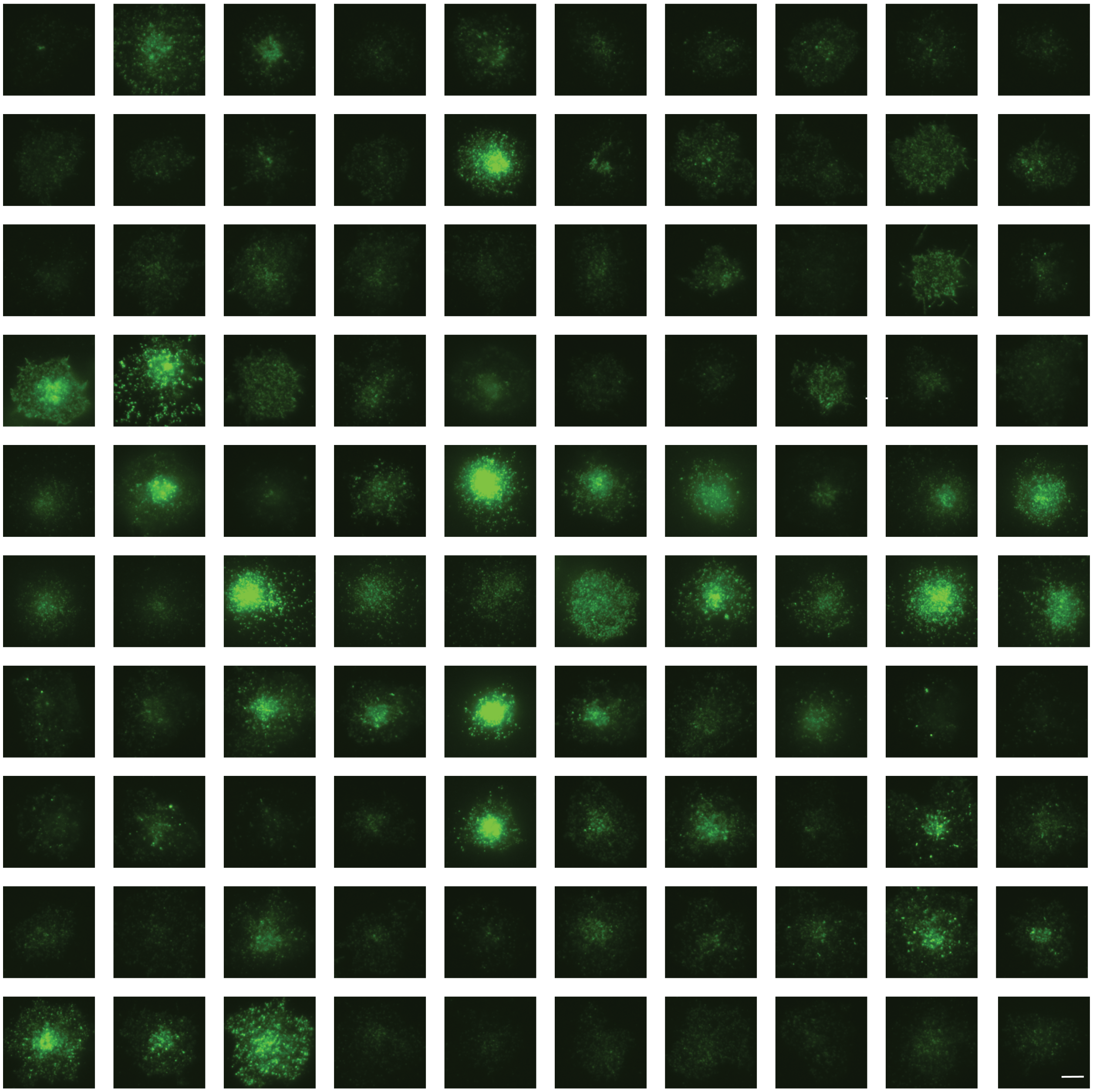

Supplemental Figure S3. All 100 TCR images from the OKT3 surfaces (*Class 1*) used to develop SCAMPI. All images were displayed in the same contrast. Scale bar: 5 µm.

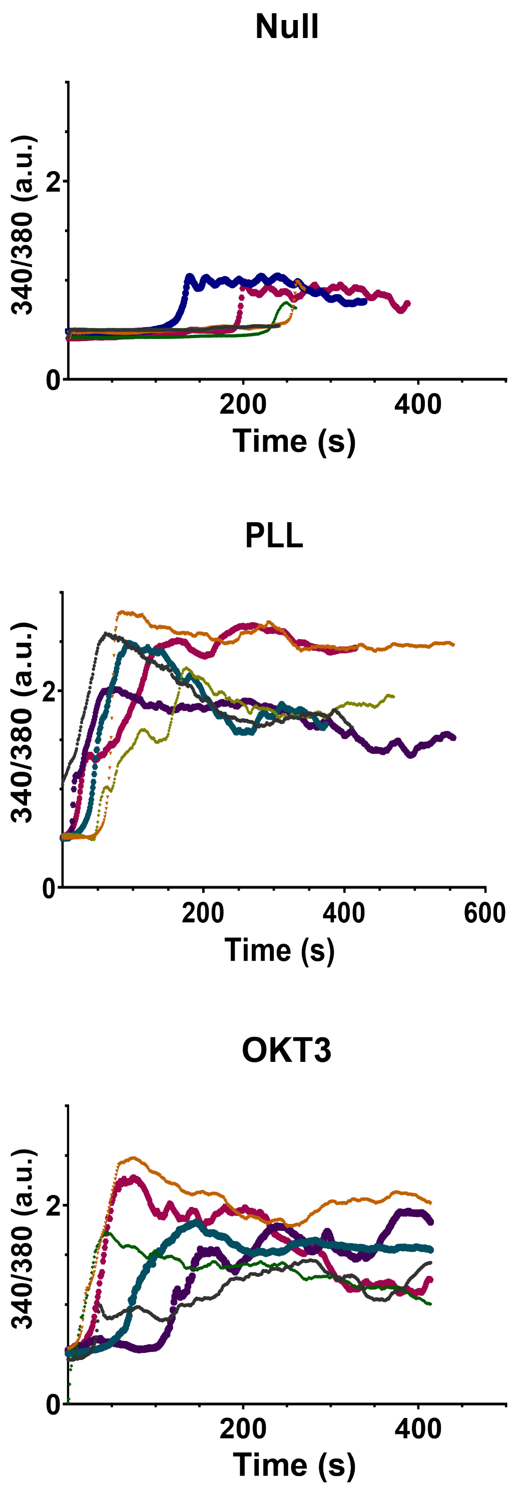

Supplemental Figure S4. Calcium imaging data from the three surface conditions: Null, PLL, and OKT3.

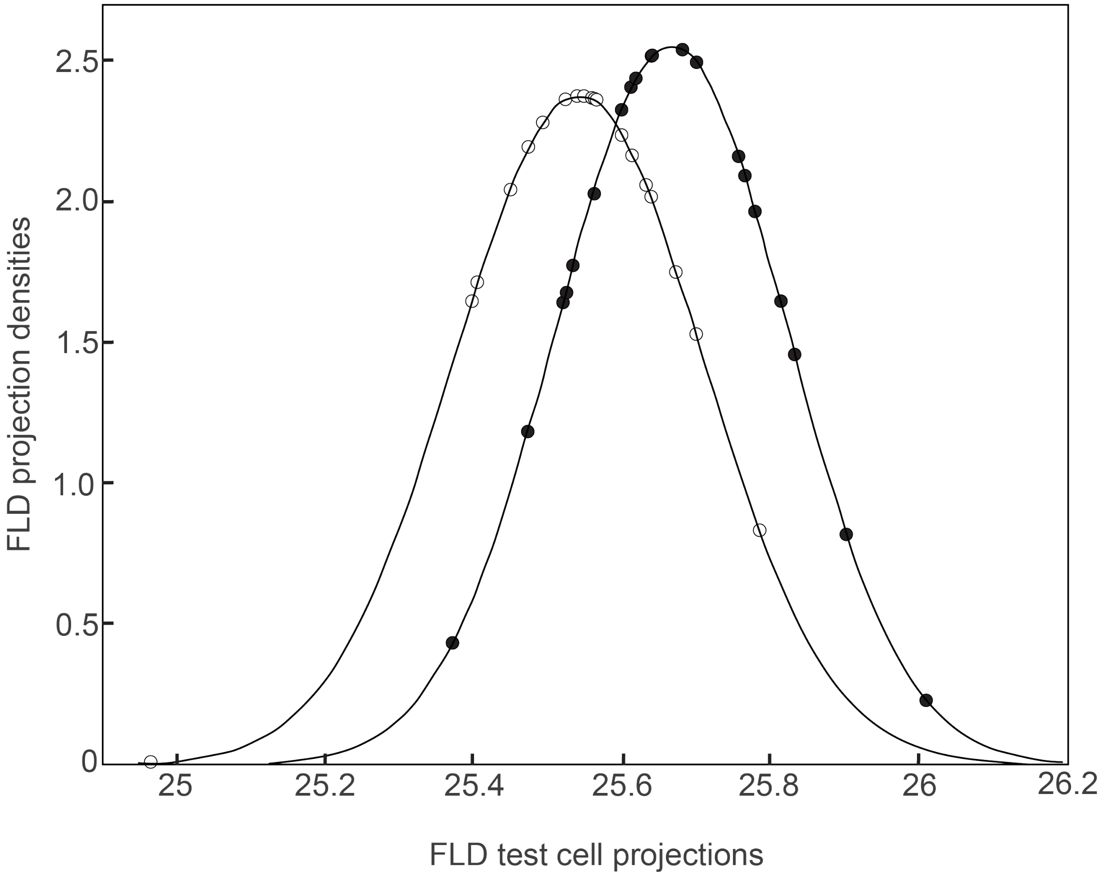

Supplemental Fig S5. FLD test cell projections for two nonoverlapping data sets from the OKT3 class.

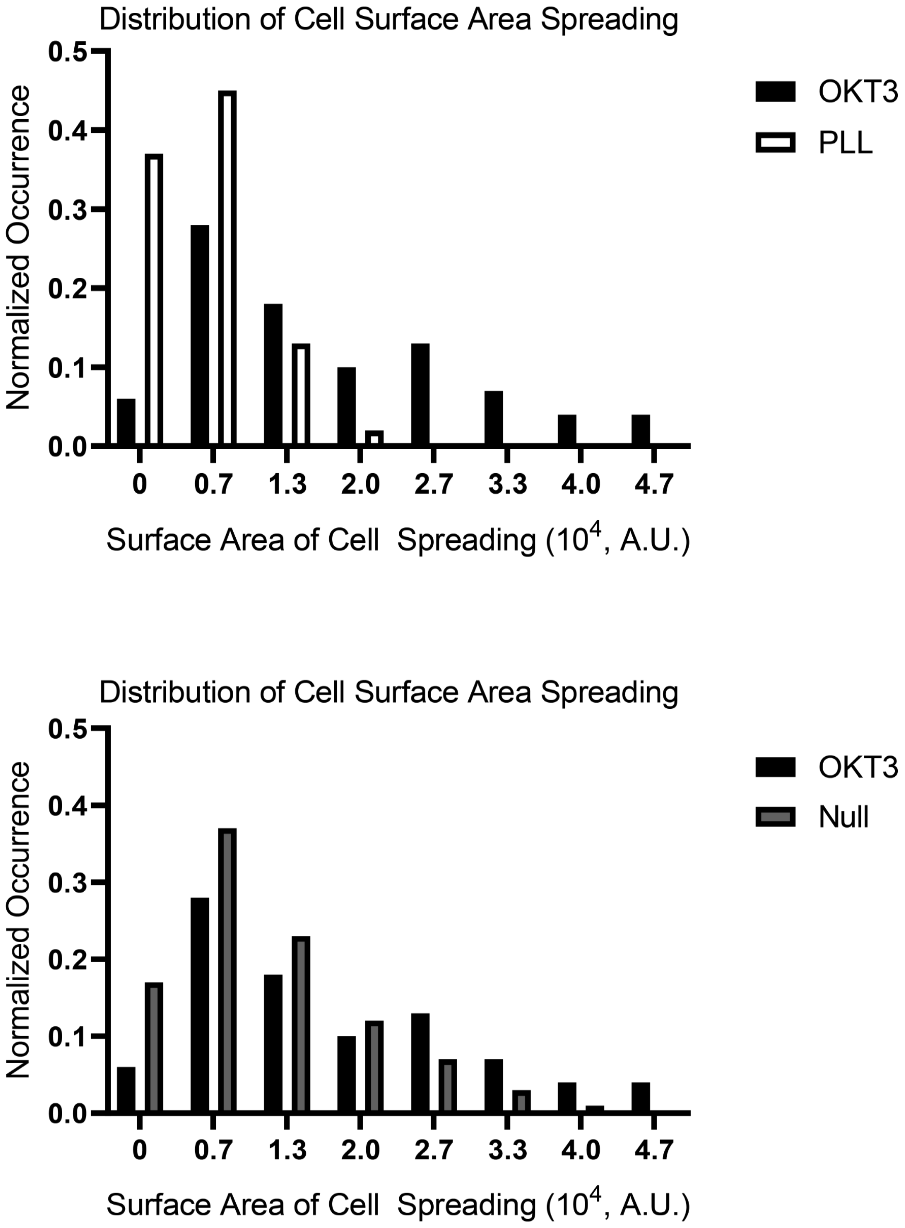

Supplemental Figure S6. Distribution of Cell Surface Area Spreading on the three different surface conditions. Cell surface areas were obtained using MATLAB.

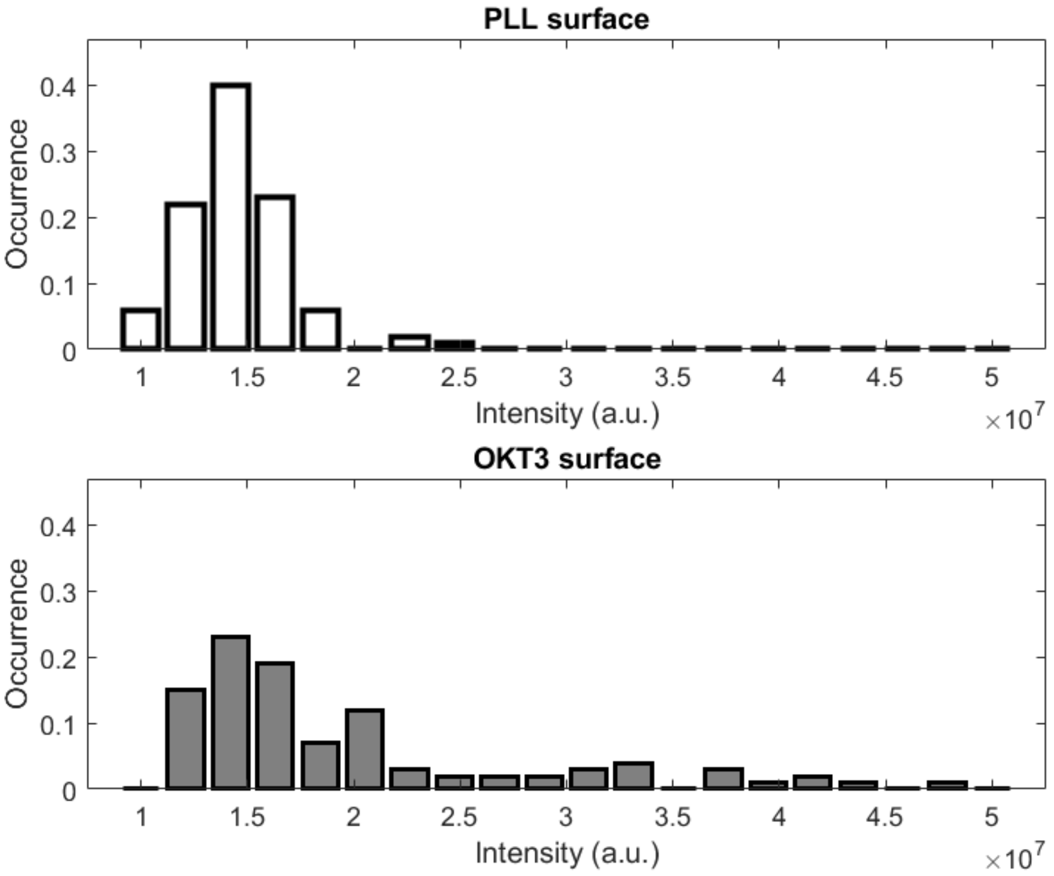

Supplemental Figure S7. Distribution averaged fluorescence intensity of TCR images on PLL and OKT3 surface conditions. Averaged fluorescence intensity of TCR images were obtained using MATLAB.

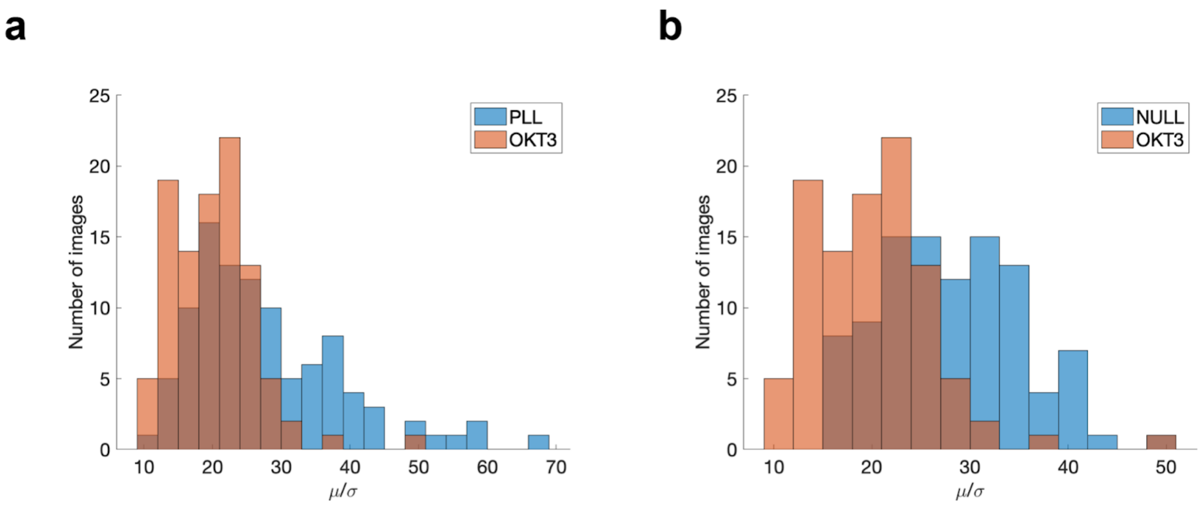

Supplemental Figure S8: Lognormal distribution fit to the pixel intensities to discriminate between PLL *vs*. OKT3 (a) and NULL *vs*. OKT3 (b) images. μ and σ represents the mean and standard deviation of the lognormal fit from each image.

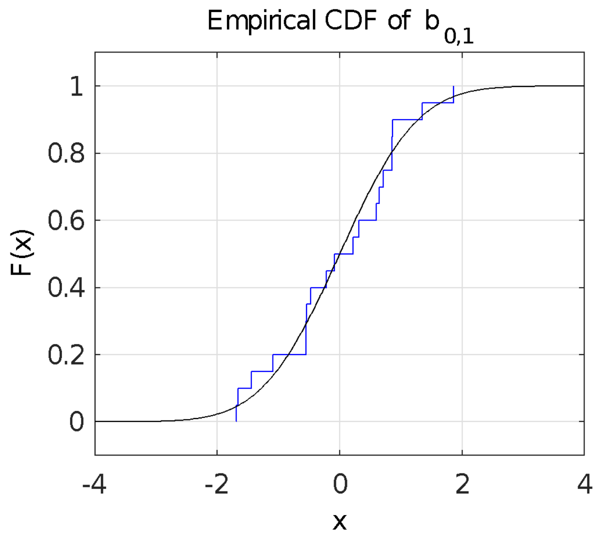

Supplemental Figure S9. Typical normality check of 20 $b_{k,l}$ parameters against a N(0,1) cumulative distribution function, x represents the variable $b_{k,l}$, F(x) represents the cumulative probability function of the random variable x.

Supplemental Note 1: Image modeling method

We hypothesize the gray-scale pixel intensity value, $v(x,y)$,of an image in Cartesian coordinates satisfies the PDE:

$$\alpha_1 \frac{\partial v}{\partial x} + \alpha_2 \frac{\partial v}{\partial y} + \alpha_3 \frac{\partial^2 v}{\partial x \partial y} = u(x,y) \tag{1a}$$

This model is the temporal equilibrium form, $\frac{\partial v}{\partial t} = 0$, of the advection-diffusion equation. Ordinarily, diffusion in general would be in the x, y, and xy directions, but x and y diffusion coefficients on the T cell membrane were uniformly insignificant in estimation and as a result were eliminated while the xy diffusion estimates were highly significant. The membrane physical shape, see the examples in **Fig. 1**, suggests a circular feature for ~~cells~~ membranes that could account for these observations.

In (1a), $u(x,y)$ is a zero mean random noise variable to be minimized in variance to estimate the model parameters. This equation has a long history as a model for a wide range of images[1,2] but also as an advective and diffusion model of particles coagulating over space and time.[3] To estimate parameters, we approximate (1a) with a partial difference equation (PdE) on a grid indexed by $x = i\Delta x$, $y = j\Delta y$ and approximate derivatives by backward differences. In our imaging experiments, Δx=Δy=110 nm which amounts to approximately 1230 pixels per mm^2. For discrete images of unit width pixels (1a) becomes the matrix equation

$$v_{i,j} = \beta_{0,1} v_{i,j-1} + \beta_{1,0} v_{i-1,j} + \beta_{1,1} v_{i-1,j-1} + e_{i,j}, \tag{2a}$$

in which $e_{i,j}$ is the spatially discrete version of $u(x,y)$. $\beta_{0,1}$ and $\beta_{1,0}$ are the advective parameters, and $\beta_{1,1}$is the diffusion parameter. The vector transform of a matrix sum is the sum of vector transforms. The vector transform of a *m by n* matrix to a *m*×*n by 1* vector is done by concatenating the matrix columns with the first column on top. Define ***q***= $vec(v_{i,j})$, **z**$_1$= $vec(v_{i,j-1})$, **z**$_2$= $vec(v_{i-1,j})$, **z**$_3$= $vec(v_{i-1,j-1})$, and $vec(e_{i,j})$= ε, $(2a)$ becomes

$$q = [z_1\ z_2\ z_3]\beta + \varepsilon = Z\beta + \varepsilon, \tag{3a}$$

in which $the\ q$ vector represents the image to be modeled, *Z* is a design matrix of spatially lagged versions of q , $\beta^T = [\beta_{0,1}\ \beta_{1,0}\ \beta_{1,1}]$, and ε is a zero mean residual error vector whose variance is minimized by the OLS estimate b of β,

$$b = (Z^T Z)^{-1} Z^T q. \tag{4a}$$

Zb is the OLS estimate of the image and $\hat{\epsilon}$ is the estimated image model error, $\hat{\epsilon}$ =*q* – *Zb*.
For the model in (2a), image pixels must be sacrificed to make *q* and *Z* compatible for addition. This data loss is not usually significant; for example, the image in **Fig. 2c** has the *samples per parameter estimated* in the OLS regression decrease from 26133 to 25761. **Table S1** shows the regression statistics for the image in **Fig. 2c**.

Table S1. Regression statistics for the image in **Fig. 2c**.

Parameters	$\beta_{0,1}$	$\beta_{1,0}$	$\beta_{1,1}$
Estimates	0.314	1.2166	-0.5340
Student *t*	41.58	79.18	-51.97
$Var(\hat{\epsilon})$ = 262.77	Var(**q**)= 74881	N = 77284	R^2 = 99.66%

The Student t statistics are computed using the White parameter covariance matrix estimate corrected for heteroskedastic and autocorrelated, residuals.[4] We found it is common for OLS image models defined by vec transformations to exhibit heteroskedastic and autocorrelated residuals, that is, the random error terms are, in all probability, from different distributions, are autocorrelated and not normally distributed. The White parameter covariance estimates are asymptotic results which compensate for these deficiencies. With 77284 degrees of freedom in this regression, such asymptotic conditions surely prevail. The extraordinarily large Student t values reflect the large degrees of freedom per estimated parameter; typical of OLS image models.

The general linear, constant coefficient PdE of 2 independent variables has a discrete PdE representation:

$$v_{i,j} = \sum_{k=0}^{p} \sum_{\substack{l=0 \\ k+l>0}}^{q} \beta_{k,l} v_{i-k,j-l} + e_{i,j} \tag{5a}$$

A regression of the vector transform of (5a) can be shown to require (p+1)(q+1) OLS parameters. This general model of order $r = max\ (p, q)$ of an m by n image requires $(r + 1)(m + n - r - 1))$ pixels to be sacrificed as in (2a) above. Note that (2a) is (5a) for $p = q = 1$.

Supplemental Note 2: Fisher Linear Discriminant

Each image has an OLS-estimated vector of parameters. Recall that the Fisher Linear Discriminator (FLD) will project individual vectors onto a line so that the variation between the projected samples is maximized relative to the variation within the projected samples. To see how this is executed with OLS image parameters, let B_1 and B_2 be the class parameter matrices of m rows (number of images) by k columns. Let G_1 and G_2 be the estimated covariance matrices of the B matrices and G_p the estimated covariance matrix of $B_p = [B_1\ B_2]^T$. Then the eigenvector v_c satisfying:

$$(G_1 - G_2 - G_p)v_c = \lambda(G_1 + G_2)v_c\,, \tag{6a}$$

for the unique eigenvalue $\lambda \neq 0$, is the optimal projection vector for discriminating cells on the OKT3 surface from the cells on the PLL surface. (6a) is solved for v_c and v_c is used to project test images for classification.

The class parameter covariance matrices, indicated as G_1 and G_2 have classification significance because the parameters are normally distributed.[7] The Kullback minimum discrimination statistic (KMDS) for covariance matrices, G=0.5(G_1+G_2), is

$$I(*,2) = 100\log(\det(G)/\det(G_1)) + 100\log(\det(G)/\det(G_2)) \quad (7a)$$

and for this application is Chi-square distributed with 6 degrees of freedom. For our estimates (7a) is 274.2. The KMDS for equalities of means is also Chi-square with 6 degrees of freedom and is estimated at 92.2. The Chi-square with 6 degrees of freedom has significance level at 4 mean values of 0.00052; at 45.6 and 15.4 times that mean value, respectively, the null hypothesis of equality is emphatically rejected for covariance matrices and expected values.

The operational test sketched in **Fig. 3a** required 160 training set OLS models and 40 test set OLS models, each with 77284 sample points, plus the solution of (6a) for v_c. This required 21.89 seconds of CPU time.

Discrimination efficacy increases with the number of parameters per image. However, constraints become tight for experiments with a relatively small number of class members. Equation (6a) only has a non-trivial solution for v_c if the number of parameters is less than or equal to the total number of class members less two.[3] A second constraint is: As $p + q$ in (5a) increases G_1, G_2, and G_p fail to be positive definite, implying the eigenvector solution of (6a) is no longer valid.[3] For the training classes of 160 total cell images samples, 160 - 2 = 158 or fewer parameters per image is a generous constraint. But the G matrices in (6a) are not positive definite for $p + q > 7$. If there are 20 images per class, then 38 parameters per image becomes a tight constraint, in addition to that of G matrix positive definiteness. For such cases, logistic regression becomes a plausible alternative classification approach (**SI Note 3**).

Supplemental Note 3: Class probability discrimination using Logistic Regression

Logistic regression as a discrimination tool estimates the probability a given image belongs to a specific class and does so without FLD-type data constraints [5]. Let i = 1,2,…40 be an image index for 20 cells on the OKT3 surface and other 20 on the PLL surface. The Bernoulli random variable Y_i is assumed to take the value y_i =1 if image i is in the class with images from OKT3 coated surface and 0 if the image is from the PLL coated surface. p_i is the conditional probability image i is in the *class with OKT3 surface* conditioned on explanatory (independent) variables hypothesized to control class membership. The design matrix of explanatory variables for the LR is [$a_o + a_1 z$] where z is the 40 FLD test projections (horizontal axis) indicated in **Fig. 3c**. Both *Class 0* and *Class 1* cell projections pass a Kolmogorov-Smirnov normality test. The hypothesized LR model uses data on y_i and z_i to estimate a_o and a_1 in the logistic regression:

$$y = (1 + \exp[-(a_o + a_1 z)])^{-1} \quad (8a)$$

The class identities are known for all images from the experiment producing the cell images: For OKT3-stimulated cells, 1 through 20, the y_i data is 1 and cells on the PLL surface, 21 through 40 have $y_i = 0$ (**Fig. 3e**). The probabilities p_i are estimated by maximizing the likelihood function of the independent Bernoulli distribution for 40 samples; this is a nonlinear optimization [5].

MLE (maximum likelihood estimator) logistic regression is ordinarily inferior to exact logistic regression (ELR) for small sample sizes, 40 in our case, but that is not true in the current application due to the precision offered in our image model parameter estimates: We estimated probability estimates for our 40 samples by ELR and they were not statistically different from the MLE estimates. A 3-parameter regression of MLE outcomes on ELR outcomes had an $R^2$of 0.9998 and a maximum residual error of 0.0914%.

The logistic regression results from (7a) are the estimated p_i and logistic regression function of **Fig. 3d**. COD* in **Table S2** is the Tjur [6] Coefficient of Discrimination, $0 \leq \text{COD} \leq 1$. With normally distributed independent variables (**Fig. S3**), the Wald statistics are asymptotically Student-*t*, so the parameter estimates are significant at level 0.0044.

Table S2. **Logistic statistics for the logistic regression in Fig. 3d.**

Parameters	a_o	a_1
Estimates	-132.0278	47.3306
Wald Statistic	-2.7529	2.7519
	**Deviance = 20.9497**	**COD* = 0.6540**

As noted in the text, one could directly use the matrices of the b vectors, B_1, B_2, in a LR to predict individual class probabilities. In this case ($a_o + a_1 z$) in (7a) is replaced by $[B_1, B_2]^T$. The result of that regression was found to be $[a_o\ a_1\ a_2\ a_3] = [59.1\ \ -66.3\ \ -54.3\ \ -51.9]$ with respective Wald statistics of [0.91 -1.06 -0.69 -0.76]. None of the estimates are significant even though the regression deviance is about the same in both regressions.

References of SI

1. Topol, E. J. High-performance medicine: the convergence of human and artificial intelligence. *Nat. Medicine* **25**, 44–56 (2019).
2. Litjens, G. *et al.* A survey on deep learning in medical image analysis. *Med. Image Anal.* **42**, 60–88 (2017).
3. Wilks, S. *Mathematical Statistics* (Wiley, New York, 1963).
4. White, H. Maximum likelihood estimation of misspecified models. *Econometrica* **51**, 513 (1983).
5. Hosmer, D. W., Jr., Lemeshow, S. & Sturdivant, R. X. *Applied Logistic Regression* (John Wiley & Sons, 2013).

6. Tjur, T. Coefficients of determination in logistic regression models—a new proposal: the coefficient of discrimination. *Am. Stat.* **63**, 366–372 (2009).
7. Kullback, S. *Information Theory and Statistics* (Dover, 1968).
